# Arthroscopic Plication of the Anterior Portion of the Annular Ligament for Lateral Elbow Instability

**DOI:** 10.1016/j.eats.2024.103403

**Published:** 2025-01-07

**Authors:** Aurelien Traverso, Simone Cassin, Frederic Vauclair, Martina Archinà, Armando Gatto, Paolo Arrigoni, Pietro S. Randelli

**Affiliations:** a Service d’orthopedie et de traumatologie, Centre Hospitalier Universitaire Vaudois (CHUV), Lausanne, Switzerland; b Scuola Di Specializzazione in Ortopedia e Traumatologia Università Degli Studi Di Milano, Milan, Italy; c Bone and Motion Center, Clinique Bois-Cerf, Lausanne, Switzerland; d Department of Surgery, Orthopaedics, Traumatology, Microsurgery and Rehabilitation, Federico II University of Naples, Naples, Italy; e Clinica Ortopedica, Azienda Socio Sanitaria Territoriale Centro Specialistico Ortopedico Traumatologico Gaetano Pini-CTO, Milan, Italy; f Laboratory of Applied Biomechanics, Department of Biomedical Sciences for Health, Università Degli Studi Di Milano, Milan, Italy; g U.O.C. 1°Clinica Ortopedica, ASST Centro Specialistico Ortopedico Traumatologico Gaetano Pini-CTO, Milan, Italy; h Research Center for Adult and Pediatric Rheumatic Diseases (RECAP-RD), Department of Biomedical Sciences for Health, Università Degli Studi Di Milano, Milan, Italy

## Abstract

The elongation of the annular ligament is a rare injury causing disability and instability of the elbow. The loosening of the annular ligament is also a pathological sign of symptomatic minor instability of the lateral elbow and could be evaluated through the loose collar sign. This Technical Note aims to describe a safe and reproducible all-arthroscopic procedure to plicate the annular ligament with suture anchor through posterolateral and midlateral portals.

The annular ligament is a strong fibro-osseous circular structure that attaches to the anterior margin of the radioulnar notch. The annular ligament is the primary stabilizer of the radial head^1^ and is part of the lateral collateral ligament complex,^2^^,^^3^ a major stabilizer of the proximal radioulnar joint.^4^

The symptomatic minor instability of the lateral elbow (SMILE) condition is characterized by intra-articular pathological findings, such as the loose collar sign, an indicator of patholaxity and instability.^5^, ^6^, ^7^ Classically, these patients experience lateral pain that becomes recalcitrant after 6 months. On clinical examination, they present with localized tenderness and positive SALT and PEPPER tests.^8^

When pain becomes recalcitrant and conservative treatments are ineffective, surgery may be necessary.^9^ The plication of the annular ligament could reduce SMILE symptoms and improve patients’ elbow functionality, especially in patients with recurrent simple elbow dislocations.

This Technical Note aims to describe an arthroscopic suture anchor technique to repair and reduce the annular ligament loosening in SMILE patients (plication of the annular ligament).

## Surgical Technique

The surgical technique is shown in Video 1 and summarized in Table 1. The main steps are described in the following section.

**Table 1:** Main Surgical Steps for Plication of the Annular Ligament Techniques

• Positioning of the patient in a modified lateral decubitus position
• Positioning of the arm at 100° of flexion and the elbow at 90° of flexion
• Placement of an arthroscope in the posterolateral portal and shaver in the midlateral portal
• Evaluation of the loose collar from the midlateral portal with a mini-spreader
• Creation of anterolateral and anteromedial portals
• Insertion of a 30° arthroscope through the anteromedial portal
• Inspection of the radial side joint
• Insertion of suture anchor through the anterolateral portal
• Passing of a shuttling suture through the annular ligament with a half-moon spectrum
• Retrieval of sutures from the anterolateral portal
• Securing of sutures with standard sliding knots
• Analysis of a negative loose collar sign

This procedure is carried out in accordance with the Code of Ethics as described by the Declaration of Helsinki.

### Patient Positioning

The patient is set in a modified lateral decubitus position, with the operative arm at 100° of shoulder flexion using an arm holder. The elbow is positioned at 90° of flexion, with the forearm hanging free to gravity. A tourniquet is positioned at the axilla and inflated to 250 mm Hg. The elbow joint is distended with 20 cc of saline solution.

### Arthroscopic Portals and Diagnostics

First, the arthroscope is inserted in the posterolateral portal (PL), created with an incision between the apex of the olecranon and the lateral epicondyle. By blunt dissection, the capsule is perforated, following the bone margin, allowing it to “fall” into the olecranon fossa. The midlateral portal (ML) is created with an incision where the soft spot is. An outside-in technique is preferred to introduce the surgical instruments parallel or slightly inclined to the radial head.

**Fig 1:**
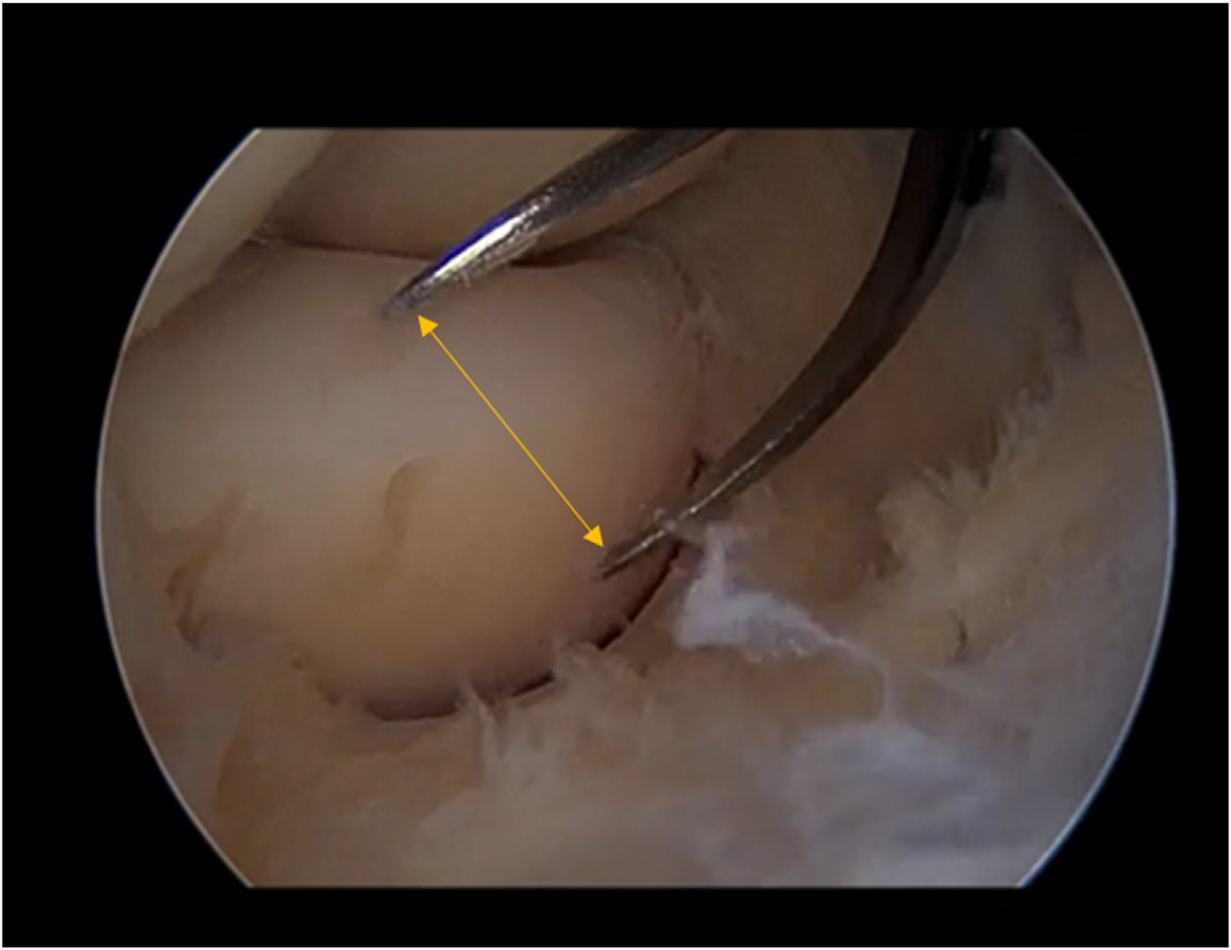
Loose collar sign assessment (yellow arrow) before annular ligament plication with a mini-spreader through the ML in a SMILE patient. Patient side: left elbow. Position: right lateral decubitus. Viewing portal: proximal anteromedial portal. (ML, midlateral portal; SMILE, symptomatic minor instability of the lateral elbow.)

The radiocapitellar joint is evaluated through the PL and ML. The loose collar sign is measured^6^ to confirm the annular ligament loosening with a mini-spreader inserted through the ML (Fig 1). The loose collar could be defined by the exposure of the radial neck beyond the cartilaginous portion of the head, with the elbow at 90° of flexion, and it can be measured during arthroscopy with a mini-spreader as the distance between the annular ligament and the top of the radial head.^6^

The surgeon creates a proximal anteromedial portal (AM) 2 to 3 cm proximal to the medial humeral epicondyle and 1 cm anterior to the intramuscular septum, incising only the skin with a blunt instrument. It is important to localize the medial intermuscular septum and gently follow it anteriorly until the front of the humeral surface is reached. When sliding on the humeral epiphysis, the instruments can reach the anterolateral joint, toward the radial head. Insertion of a 30° arthroscope from the AM allows intra-articular diagnostic evaluation of the anterior compartment. Finally, the anterolateral portal (AL) is created, 1.5 to 2 cm proximal and 1 cm anterior to the lateral epicondyle; for proximity to the radial nerve, we recommend creating this portal by an outside-in technique, controlling the position from the PL.

Through the AL, the surgeon can inspect the radial side of the joint.

### Anterior Plication Steps

**Fig 2:**
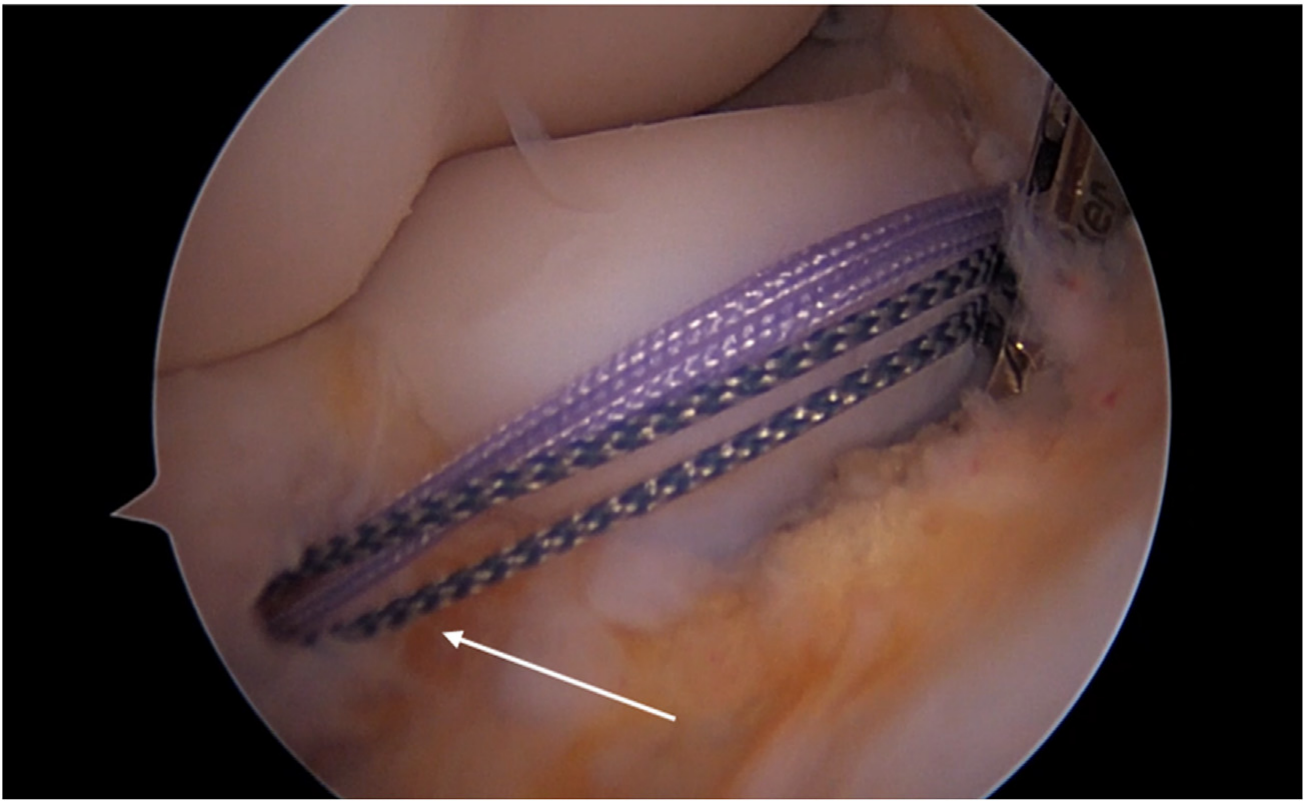
A suture anchor is inserted on the lateral side of the coronoid process, at the level of the anterior annular ligament (white arrow). Patient side: left elbow. Position: right lateral decubitus. Viewing portal: proximal anteromedial portal.

The scope is inserted through the AM, offering a clear view of the radioulnar joint, while instruments and anchors are passed through the AL. A suture anchor (Lupine Anchor; DepuySynthes) is inserted on the lateral side of the coronoid process at the level of the anterior annular ligament margin of the radial notch^1^ (Fig 2).

**Fig 3:**
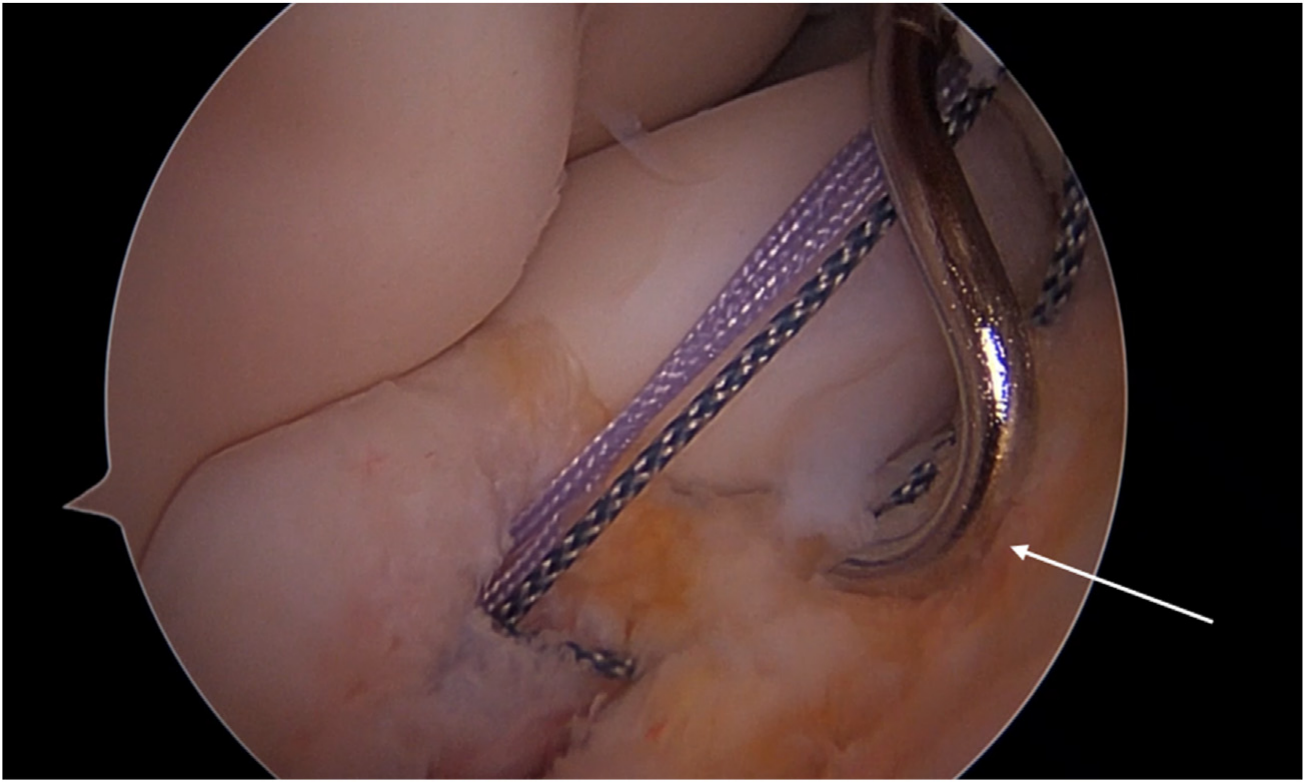
A half-moon spectrum is used for passing a suture through the annular ligament from inside to outside and from lateral to medial to limit the risk of posterior interosseous never injury (white arrow). Patient side: left elbow. Position: right lateral decubitus. Viewing portal: proximal anteromedial portal.

The creation of a more proximal lateral portal is useful to obtain a more adequate angle of approach for the anchor placement. Sutures are passed through the annular ligament with a half-moon spectrum from inside to outside and from lateral to medial to limit the risk of posterior interosseous never injury (Fig 3).

Sutures are retrieved from the AL and secured 2 by 2 with standard sliding knots. Thereafter, the annular ligament is plicated back to the bone. The surgeon could measure the loose collar to confirm the annular ligament reparation.

### Rehabilitation

After surgery, the limb should be protected with an arm brace for approximately 2 weeks. Gradual recovery of the range of motion is initiated until fully achieved, usually within an additional 3 weeks. Thereafter, strengthening can be initiated. Wounds are treated routinely. Throwing and overhead sports should be limited up to 6 months after surgery.

## Discussion

We describe a safe and reproducible arthroscopic technique to reduce annular ligament loosening in SMILE patients.

Elbow arthroscopy in recent years has become a much more common procedure, even though it remains challenging, especially for less experienced surgeons. The advantage of an arthroscopic approach for annular ligament loosening is initially diagnostic, smaller incisions with less soft tissue dissection, better visualization of the joint, and direct evaluation of final retensioning on the annular ligament compared to open surgery, which enables the identification of annular ligament injuries (Table 2).

**Table 2:** Pearls and Pitfalls of the Plication of the Annular Ligament Techniques

Pearls	Pitfalls
Precise diagnosis	Risk of damage to median, ulnar, and radial nerves
Minimally invasive	Risk of damage to the cartilage
Tissue sparing	Risk of musculotendinous entrapment in the suture
Easily defined anatomic landmarks	Risk of stiffness
Direct visualization of the result of the repair	Plication success could be influenced by tissue quality
Time saving	Needs to be familiar with elbow arthroscopy
Highly anatomic	Risk of coronoid fracture

Lateral collateral ligament complex repair has previously been described,^6^^,^^9^ but this procedure focuses on the plication of the annular ligament and its retensioning, a fundamental part of SMILE treatment, confirmed by the negativization of the loose collar sign.

A systematic approach to addressing the elbow joint during an arthroscopic procedure allows the surgeon to decrease the complication rates and time of surgery.^10^ The risks are the standard ones for elbow arthroscopic procedures^11^ and, more specifically, are related to portal management. While creating the ML, it is important to enter toward the radioulnar joint instead of the radiocapitellar joint to reduce the risk of iatrogenic injury to the articular cartilage.^6^ It is mandatory to avoid the intermuscular septum because of the risk of injury to the ulnar nerve while establishing the AM; it is also very important to slide under the brachialis muscle to protect the median nerve and vascular structures during instrument introduction.^6^ The AL is the portal most exposed to iatrogenic lesions because of its proximity to the radial nerve and joint capsule.^6^

Suture management must be performed with care to avoid musculotendinous entrapment (Table 2).

The plication of the annular ligament arthroscopic technique provides surgeons with a fast, reproducible, and safe way to address annular ligament loosening. In our experience, it can be used effectively to improve stability and decrease pain in SMILE patients, without damaging the elbow joint.

## Disclosures

The authors declare the following financial interests/personal relationships which may be considered as potential competing interests: A.T. received financial support for his fellowship from Bonebridge and SICPA. All other authors (S.C., F.V., M.A., A.G., P.A., P.S.R.) declare that they have no known competing financial interests or personal relationships that could have appeared to influence the work reported in this paper.

## Supplementary Data

Video 1 Arthroscopic plication of the anterior portion of the annular ligament. The elongation of the annular ligament is a rare injury causing disability and instability of the elbow. The arthroscopic elbow annular ligament plication provides surgeons with a fast, reproducible, and safe way to address annular ligament loosening. Patient side: left elbow. Position: right lateral decubitus. Viewing portal: proximal anteromedial portal.
